# Analysis of Candidate Genes for Growth and Milk Performance Traits in the Egyptian Barki Sheep

**DOI:** 10.3390/ani10020197

**Published:** 2020-01-23

**Authors:** Ibrahim Abousoliman, Henry Reyer, Michael Oster, Eduard Muráni, Mosaad Mourad, Mohamed Abdel-Salam Rashed, Ismail Mohamed, Klaus Wimmers

**Affiliations:** 1 Institute of Genome Biology, Leibniz Institute for Farm Animal Biology, Wilhelm-Stahl-Allee 2, 18196 Dummerstorf, Germany; 2 Department of Animal and Poultry Breeding, Desert Research Center, 1 Mathaf El-Matareya st, 11753 El-Matareya, Cairo, Egypt; 3 Faculty of Agriculture, Ain Shams University, Hadayek Shobra, 11241 Cairo, Egypt; 4 Faculty of Agricultural and Environmental Sciences, University of Rostock, Justus-von-Liebig-Weg 7, 18059 Rostock, Germany

**Keywords:** Barki sheep, growth traits, milk performance, single nucleotide polymorphism, association analysis

## Abstract

**Simple Summary:**

The Barki sheep breed is one of the main sheep breeds in Egypt, and it is well adapted to the harsh desert conditions in the Mediterranean zone. Growth performance has an important role regarding the supply of red meat for human consumption. In addition, milk production is very important to adequately feed newborn lambs and prevent them from starvation. In this study, segregating single nucleotide polymorphisms (SNP) were identified in the coding regions of eight candidate genes for growth and milk traits by direct sequencing. Subsequently, a population of Barki ewes and lambs was screened for these SNPs, and associations between genotypes and traits of interest were assessed. Out of the candidate genes, SNPs of *LEP, STAT5A, PRL*, and *GHRHR* were significantly associated with phenotypes. This study provides first insights into the genetics of milk and growth traits in the Barki sheep. The findings concluded that *LEP, STAT5A, PRL*, and *GHRHR* might be regarded as candidate genes to improve the Egyptian Barki sheep breed.

**Abstract:**

The most common sheep breeds of Egypt are Ossimi, Rahmani, and Barki breeds. The latter one is well adapted to the challenging desert environment, characterized by food shortage and a high temperature fluctuation. Growth performance of Barki sheep has an important economic value in terms of minimizing the shortage of mutton meat in Egypt. Further, milk production is of great importance for feeding newborn lambs. Eight candidate genes, recently associated with production traits in different breeds, were used to study the effect of genotype on lamb growth and ewe milk traits. The examined genes were *LEP, IGF1, DGAT1, STAT5A, PRL, CSN1S2, GHR,* and *GHRHR*, of which one representative single nucleotide polymorphism (SNP) located in the coding region was selected for genotyping. Data from 251 Barki sheep were used in this study. Association analysis between SNPs and lamb growth traits identified rs420693815 of the *LEP* gene to be significantly associated with weaning weight and average daily gain. In ewes, significant effects on milk yield and composition have been estimated for *LEP* (rs420693815), *STAT5A* (rs161082816), *PRL* (rs422713690), and *GHRHR* (rs414991449). The results indicated that these genes might be considered as interesting candidates for further investigations to improve growth and milk performance in Barki sheep.

## 1. Introduction

Small ruminants play an important role in supplying meat and meat products in arid regions. Regarding the Egyptian agriculture sector, sheep production serves as a valuable source of income to farmers and as an important source of meat and milk [1]. In Egypt, the contribution of sheep is about 6.4% of the total red meat production. The most common indigenous sheep breeds in Egypt are Ossimi, Rahmani, and Barki [2]. The sheep population in Egypt has increased rapidly during the last five years, reaching about 5.7 million animals in 2017 [3]. Barki sheep spread along the northwestern coastal zone (NWCZ) of Egypt with a population of 470,000 heads (8.5% of the total Egyptian sheep population). Barki sheep are raised under the transhumant system, grazing grasses, little bushes, and plants that grow during the period between August and June as a result of the rainfall of this Mediterranean area. Barki sheep are well adapted to harsh desert conditions, such as feed shortage and high ambient temperatures and have the ability to produce a considerable amount of meat, wool, and milk under these conditions [4]. However, the newborn lambs suffer from the starvation because of the scrawny milk production of their dams. This problem augments lamb mortality particularly in the early ages. Hence, there is a great interest in understanding the genomic architecture of growth and milk traits of these animals in order to improve both meat and milk characteristics. Improvement programs depending on genetic information should be established aiming to facilitate the selection of breeding animals, which will actually improve these important traits. In various sheep breeds, researchers have genotyped such molecular markers in order to study the association of candidate genes and milk performance traits (e.g., some milk traits in Sfakia sheep [5] and carcass traits in Iranian Mehraban sheep [6]). Highly important candidate genes, which showed association with milk and growth traits, are prolactin (*PRL*), leptin (*LEP*), insulin-like growth factor hormone 1 (*IGF1*), diacylglycerol O-acyltransferase 1 (*DGAT1*), signal transducer and activator of transcription 5 (*STAT5*), alpha (α)s2-casein (*CSN1S2*), growth hormone receptor (*GHR*), and growth hormone-releasing hormone receptor (*GHRHR*) [7,8,9]. Prolactin is essential for lactation and plays an important role in milk production [10]. Polymorphisms in *PRL* can be used as a candidate marker associated with milk yield and milk composition traits [7]. Leptin is a non-glycosylated protein, which plays an important role in animal growth and metabolism. It regulates feed intake, energy metabolism, and fat distribution in the body [11]. It has been shown that leptin influences milk performance in cattle [12] and in Murrah buffaloes [13]. As a member of the *IGF* family, *IGF1* is considered an important factor associated with cell differentiation, embryogenesis, metabolism [14,15], reproduction, and fetal development [16,17]. Therefore, it is a major candidate gene for most of the productive and economic traits in sheep. *DGAT* plays an important role in triacylglycerol biosynthesis as well as milk and growth traits [18]. The *STAT* family has seven members (*STAT1-4, STAT5A, STAT5B,* and *STAT6*), and *STAT5* is known to play a central role in signal transduction from prolactin to milk protein genes [19]. Caseins represent about 80% of proteins in ruminant milk [20]. The casein family consists of four genes as a cluster (alpha (α)s1-casein, alpha (α)s2-casein, beta (β)-casein, and kappa (κ)-casein) [21,22]. *CSN1S2* alleles are associated with a normal (α) s2-casein synthesis level [23]. The growth hormone receptor initiates several signaling processes regulating body growth. As such, *GHR* and *GHRHR* are valuable candidate genes. Polymorphisms in *GHR* are associated with traits related to growth performance, body size, and meat quality in cattle [24,25,26,27]. The genetic analysis of the Barki sheep has so far been limited mainly to the assessment of some candidate genes for wool traits [28]. Concerning growth characteristics, there are only analyses available which examine the effects of *FABP4* and *calpastatin* on some carcass traits of Barki lambs [29]. Heritabilities of some growth traits in Barki sheep are 0.19, 0.20, and 0.18 for birth weight, weaning weight, and average daily gain, respectively [30]. Therefore, the current study aims to identify segregating polymorphisms of major candidate genes for growth and milk traits in a Barki population of ewes and lambs. Subsequently, a SNP trait association analysis was performed to investigate the connection between genotype and production phenotypes as a prerequisite to improve performance parameters in Barki sheep.

## 2. Materials and Methods 

### 2.1. Animals and Management

Samples and data were collected from the farm of the Matrouh Resources Project (location 1) and the Maryout research station (location 2) that belongs to the Desert Research Centre (DRC), Ministry of Agriculture, Egypt. The experiment was carried out according to all ethics and animal rights (DRC) considering all regulations in conformity with the European Union Directive for the protection of experimental animals (2010/63/EU).

Phenotypic and genotypic data from 111 Barki ewes and 140 of their lambs (44 ewes and 66 lambs from location 1 and 67 ewes and 74 lambs from location 2) from one breeding season were used in this study. Ewes were randomly mated with certain rams of the same breed according to the normal farm practice. No records were available for the rams. Animals were kept under an intensive production system and were housed in semi-open yards throughout the experimental period. The lambs were kept all day with their dams for suckling until weaning at three months of age, respecting the natural ewe−lamb relationship. Some of the examined lambs were not the offspring of the ewes under study. Furthermore, some lambs have no data for their mother’s milk. Ewes in the two locations were fed daily on a feed concentrate (0.75 kg per head) and clover hay (0.5 kg per head) during the experimental period, and lambs were fed daily only on their dam’s milk from birth to weaning age at three months of age. Fresh water was available to sheep ad libitum.

### 2.2. Phenotypic Data

Live body weight for each lamb was recorded at birth and weaning by electronic balance. Average daily gain was calculated for every lamb. Milk yield was recorded biweekly from the time of parturition until weaning using hand milking technique. Lambs were separated from their dams 12 h before milking. The ewe was milked in the morning; another milking was carried out in the evening by the same approach. Milk yield per day was measured in mL by summation of the morning and evening milking. Total milk yield was calculated by summation of the daily milk yields for 90 days. Milk samples were stored at −20 °C and chemically analyzed to determine the percentages of fat, protein, lactose, and total solids using milko-scan (130 A/SN. Foss Electric, Hillerod, Denmark).

### 2.3. Blood Samples and DNA Extraction

Blood samples were collected from the jugular vein using test tubes containing disodium ethylene diamine tetra acetic acid (EDTA-Na2). All blood samples were stored at −80 °C until DNA extraction. DNA was extracted by using a commercially available kit according to the manufacturer’s instructions (G-spin™ Total DNA Extraction kit; iNtRON Biotechnology, Seoul, Korea). 

### 2.4. Detection of Polymorphisms and Genotyping

Pooled DNA samples were prepared from five lamb samples and five ewe samples from different locations. The pooled samples were subjected to a polymerase chain reaction (PCR) to amplify specific regions of the candidate genes and to identify segregating single nucleotide polymorphisms (SNPs). The PCR assay was performed using respective primer sets in a total volume of 20 µL according to the manufacturer’s instructions (SupraTherm Taq, GeneCraft, Lüdinghausen, Germany). Gene-specific primers were designed with Primer3 software (v.0.4.0) (http://bioinfo.ut.ee/primer3-0.4.0/) according to the latest sheep genome information (Ensembl Oar_v3.1, Build v96). The primer pairs used to detect SNPs are shown in Table 1. PCR products were separated on a 2% agarose gel and visualized under UV light. For all SNPs investigated, primer pair combinations resulting in only one specific amplification signal were selected. The PCR products were purified using beads purification method (Agencourt AMPure XP, Beckman Coulter, Krefeld, Germany) and sequenced on an ABI 3500 Genetic analyzer (Applied Biosystems, Foster City, CA, USA). Sequencing results were aligned, and the SNPs were detected using Bio Edit software (V 7.0.5.3). Subsequently, all animals with phenotypic data were genotyped by Kompetitive Allele Specific PCR (KASP, LGC Genomics, Teddington, Middlesex, UK). KASP assays were developed for corresponding SNPs, validated in a subset of samples, and applied to the entire sample set. The PCR mixture consisted of 10 μL according to the manufacture instructions. The PCR products were amplified and analyzed using a Light Cycler 480 machine (Roche, Mannheim, Germany) to identify genotype clusters. 

### 2.5. Statistical Analysis 

The Hardy–Weinberg equilibrium (HWE), polymorphic information content (PIC), heterozygosity (He), and homozygosity (Ho) were tested for all alleles by using the Cervus (V3.0.7.0) program [31]. The association analysis between the SNPs of the candidate genes and phenotypes of the studied sheep traits was carried out using the general linear model (GLM) of the analysis of variance (ANOVA) by SPSS V20 (IBM, New York, NY, USA). The statistical model for ewe milk traits used was as follows: Y_ijk_ = μ + G_i_+ L_j_+ e_ijk_, where Y_ijk_ is the analyzed trait, μ is the overall mean, G_i_ is the effect of genotype (i = 3 levels, except rs409119650 of *DGAT1* gene where i = 2 levels), L_j_ is the effect of location (j = 2 levels), and e_ijk_ is the error effect. Another model was used to detect the effect of genotype on the lamb growth traits as follows: Y_ijkl_= μ + G_i_+ L_j_+ S_K_+ e_ijkl_, where Y_ijkl_ is the analyzed trait, μ is the overall mean, G_i_ is the effect of genotype (i = 3 levels), L_j_ is the effect of location (j = 2 levels), S_k_ is the effect of lamb sex (k = 2 levels), and e_ijkl_ is the error effect. *p* < 0.05 was considered significant. *p* < 0.1 was considered a tendency for significance. 

## 3. Results

### 3.1. Phenotypic Data of Growth and Milk Traits

Descriptive statistics of total milk yield in three months and percentages of milk components including fat, protein, lactose, and total solids are shown in Table 2. An overview of growth traits comprising birth weight, weaning weight, and average daily gain of Barki lamb is shown in Table 3.

### 3.2. Genetic Parameters 

Sequencing results of DNA pooled samples from ewes and lambs revealed segregating SNPs in the studied genes. One SNP was selected from every gene for subsequent genotyping. The selected SNPs were rs420693815 in exon 3 of *LEP*, rs400398060 in exon 3 of *IGF1*, rs409119650 in exon 9 of *DGAT1*, rs161082816 in exon 11 of *STAT5A*, rs422713690 in exon 3 of *PRL*, rs420391387 in exon 8 of *CSN1S2*, rs413776054 in exon 10 of *GHR*, and rs414991449 in exon 13 of *GHRHR*. Results of Hardy–Weinberg equilibrium for all selected SNPs are shown in Table 4. All selected SNPs were in Hardy–Weinberg equilibrium status (*p* > 0.05). rs409119650 and rs161082816 were in low polymorphic information content status (PIC < 0.25), while rs420693815, rs400398060, rs422713690, rs420391387, rs413776054, and rs414991449 were in moderate polymorphic information content status (0.25 < PIC < 0.50). The homozygosity of all loci sites was higher than the heterozygosity, except for rs420391387 and rs422713690, where the homozygosity was equal to the heterozygosity. In lambs, the selected SNPs for all candidate genes were in Hardy–Weinberg equilibrium status (*p* > 0.05; Table 5). rs409119650 and rs413776054 were in low polymorphic information content status (PIC < 0.25), while rs420693815, rs400398060, rs161082816, rs422713690, rs420391387, and rs414991449 were in moderate polymorphic information content status (0.25 < PIC < 0.50). The homozygosity of all loci was higher than the heterozygosity.

### 3.3. Association of SNPs with Milk Traits of Barki Ewes

Table 6 shows the results of the association analysis of SNP with milk traits of ewes. rs420693815 of *LEP* showed a trend for milk yield and fat percentage (*p* ≤ 0.1). Ewes with TT genotype had a higher milk yield than ewes with GT and GG genotypes. Ewes carrying the GT genotype had a higher fat percentage than ewes with GG and TT genotypes. For rs161082816 of *STAT5A*, the milk of ewes with AA genotype were significantly (*p* ≤ 0.05) higher in lactose percentage compared to ewes with GG and AG genotypes. For rs422713690 of *PRL*, a significant association with milk yield (*p* ≤ 0.1) was observed. Ewes with GG genotypes had a higher milk yield than ewes with AG and AA genotypes. rs414991449 of *GHRHR* was significantly associated with total solids percentage (*p* ≤ 0.05) and with protein percentage (*p* ≤ 0.1). Ewes having TT genotypes had higher total solids and protein percentages than ewes with CT and CC genotypes.

### 3.4. Association of SNPs with Growth Traits of Barki Lambs

The results of the association analysis of SNPs with lamb growth traits are summarized in Table 7. The analysis revealed rs420693815 of *LEP* as significantly associated with weaning weight and average daily gain (*p* ≤ 0.1). Lambs with GT and GG genotypes had a higher weaning weight and average daily gain than lambs with TT genotype. The other selected SNPs in the candidate genes showed a non-significant association with growth traits (*p* ≥ 0.1).

## 4. Discussion

In this study, a representative SNP of each of the selected functional candidate genes was associated with growth and milk production traits obtained from Barki lambs and ewes. Genotyping results showed that none of the selected SNPs deviates from HWE. These results indicated for the absence of strong selection pressures, probably due to the coherent housing environment and the lack of artificial selection. These facts might contribute to a stability of allelic and genotypic frequency for a long time. Results of the polymorphic information content state and homozygosity-to-heterozygosity relationships confirmed that an inbreeding scheme was applied at the different locations creating a high genetic variation between populations and lower genetic variation between individuals in the same population. These results suggest that an application of selection employing genomic information will be effective in the respective population. However, the relatively low sample size, due to the lack of management with breeding programs and routine sampling in the Barki sheep, represents a certain limitation for the genetic evaluation in this study. The results of association of SNPs with lamb growth traits showed that rs420693815 of *LEP* were significantly associated with weaning weight and average daily gain. Interestingly, rs420693815 had also a significant effect on milk traits comprising milk yield and fat percentage in the Barki ewes. The results indicated the inverse relationship between milk yield and fat percentage. Ewes with the highest milk yield had the lowest fat percentage [33]. Accordingly, lambs whose mothers had the highest fat content in their milk had a higher weaning weight and a higher average daily gain. *LEP* is considered as one of the candidate genes affecting body fat content [11]. Through signaling to the hypothalamus, leptin mediates the balance between feed intake and energy expenditure [34,35]. Due to its lipolytic effect and the regulation of fat stores, genetic variants of *LEP* might be of relevance in mobilizing lipids for, e.g., milk production with possible implication on the offspring’s body weight. In agreement, genetic variants of leptin have been shown to influence milk performance in cattle [36]. A *LEP* polymorphism was found to be significantly associated with milk yield in Najdi ewes of Saudi Arabia [37]. Moreover, several studies indicated the role of *LEP* in growth traits [38,39]. With respect to the results of the current study, it is questionable if *LEP* (rs420693815) is causative for the effects or acts as tagging SNP in linkage disequilibrium with the causal one. However, *LEP* as candidate gene might be further considered as a locus for improving performance and production traits in the breeding programs of Barki sheep. Furthermore, analyzed SNPs in *STAT5A, PRL,* and *GHRHR* revealed a significant association with milk production traits in the Barki ewes. For *PRL* (rs422713690), animals with the heterozygous AG genotype showed lower milk yields than homozygous animals. *PRL* is a hormone released from the anterior pituitary gland and acts to initiate and maintain lactation [40]. The *PRL* gene is located on the ovine chromosome 20 where putative quantitative trait loci for milk yield, fat, and protein percentage are located [41,42]. Indeed, a polymorphism in *PRL* has been shown to affect all these traits in Serra da Estrella sheep [43] and milk yield in East Friesian sheep [8]. Consequently, *PRL* might act as a marker gene for milk production traits also in the Barki sheep. Regarding the investigated SNP in *STAT5A* (rs161082816), Barki ewes which carried A alleles showed higher milk lactose percentages compared to animals exhibiting G alleles. *STAT5A* is a key player in mammary gland development [44]. In particular, *STAT5A* is known to mediate *PRL* and *GH* signals via transcriptional stimulation of gene expression in milk-secreting mammary epithelial cells. Due to its prominent role in milk production traits, *STAT5A* has been previously investigated in cattle and goat and genetic variants have been associated with milk fatty acid profiles and milk yield [45,46,47]. The significant association with milk lactose percentage emphasizes *STAT5A* as a promising candidate gene for further analyses of milk traits in Barki sheep. The SNP located in *GHRHR* (rs414991449) was significantly associated with the percentages of total milk solids and milk protein, whereby the appearance of the T allele prompted the highest values. In fact, *GHRHR* mediates effects of its ligand growth hormone-releasing hormone (*GHRH*) to regulate growth hormone (*GH*) synthesis and secretion [48,49,50]. Genetic variants in the functional candidate *GHRHR* might therefore impact on *GH* axis signaling as it has been shown for body growth in humans and mice [51,52]. In studies on sheep, the *GH* locus has been associated with milk traits such as milk fat percentage and milk yield [53,54]. Corresponding effects might be mediated via *GHRHR* on *GH* signaling affecting milk production and composition. Results did not support any significant associations of sequence variants of *IGF1*, *DGAT1, CSN1S2,* and *GHR* with growth or milk traits in the studied Barki population. This might also be related to the fact that for some of the SNPs, a low representation of alternative homozygotes was found in the studied population. However, associations of segregating SNPs in *IGF1* and *DGAT1* with growth, milk, and wool performance traits have been described in various sheep breeds such as Makeoi, Baluchi, Hu, Sarda, and Mehraban [55,56,57,58,59,60,61,62]. These breed differences might be due to artificial selection pressures or housing due to geographical conditions. Clearly, comprehensive approaches including a holistic genomic evaluation are needed to elucidate the genetics and to improve milk and performance traits of Barki sheep.

## 5. Conclusions

A SNP-trait association analysis was performed to study the effect of genotype on growth and milk performance traits in Egyptian Barki sheep. Results concluded that the selected polymorphisms in *LEP, STAT5A, PRL,* and *GHRHR* were significantly associated with lamb growth and ewe milk traits, while *IGF1, DGAT1, CSN1S2,* and *GHR* genes showed no significant associations. *LEP, STAT5A, PRL,* and *GHRHR* might be considered as interesting candidate genes for further investigations to improve growth and milk performance in the Barki sheep.

## Figures and Tables

**Table 1:** Primer pairs of the studied candidate genes.

Gene Name	Gene ID	Allele ^2^	Primer Sequence	Product Size (bp)	Annealing Temperature (°C)
*LEP* ^1^	ENSOARG00000002407	T/G(V181L)	F:AGGAAGCACCTCTACACTC R:CTTCAAGGCTTCAGCACC	471	53
*IGF1*	ENSOARG00000015856	G/A	F:GTTCTGGAATGGCAGGTTTG R:GCCACTGTCTTTGGATTTTCTC	570	60
*DGAT1*	ENSOARG00000014070	T/C	F:ACTGTGCTTCAGGGTGTCGG R:GAGTGATGGACTCTAGGAGGAAGG	429	60
*PRL*	ENSOARG00000009137	A/G	F:TGGAATTTAGATGACAAGCAACTG R:AATTGGTGGCTCAAGTGGTG	745	63
*CSN1S2*	ENSOARG00000010683	A/C	F:CCCTGAAGGAATCTGCTGAAG R:AGCCAAGCAAAATGATATAGAAGC	855	63
*GHR*	ENSOARG00000008837	C/T(P448S)	F:TGATGACCCTGATGAGAAGACTG R:TTTTGTTCAGTTGGTCTGTGCTC	857	63
*GHRHR*	ENSOARG00000007636	C/T	F:TTGTTCTTGGAGGTGAGGACTG R:AACACGGGTGGCTCTCTTG	759	63
*STAT5A*	ENSOARG00000000809	G/A	F:GGGTGCATACAGGACAGTGC R:CCAGTCTCTGGCTTTCCCAA	446	60

**^1^** primer design according to [32]; **^2^** If present, consequences at the protein level are shown.

**Table 2:** Descriptive statistics of ewe milk traits.

Trait	N	Mean	Standard Deviation	Minimum	Maximum
Total Milk Yield (kg)	111	28.95	12.64	9.90	77.40
Fat (%)	111	4.30	1.74	1.00	9.60
Protein (%)	111	5.11	1.35	2.70	9.50
Lactose (%)	111	6.34	1.42	0.81	9.90
Total Solids (%)	111	18.72	5.38	12.14	34.70

**Table 3:** Descriptive statistics of lamb growth traits.

Trait	N	Mean	Standard Deviation	Minimum	Maximum
Birth Weight (kg)	140	3.71	0.58	2.42	5.04
Weaning Weight (kg)	140	13.83	3.89	5.15	28.80
Average Daily Gain (kg/day)	140	0.112	0.04	0.02	0.27

**Table 4:** Genetic parameters of the single nucleotide polymorphisms (SNP) markers of the studied candidate genes in the Barki ewe population.

Gene	SNP Locus	Genotype	Genotypic Frequency	Allele	Allelic Frequency	He	Ho	PIC	HWE Test (*p* Value)
*LEP*	rs420693815	TT	0.15	T G	0.41 0.59	0.48	0.52	0.37	0.35
		GT	0.53						
		GG	0.32						
*IGF1*	rs400398060	GG	0.47	G A	0.69 0.31	0.43	0.57	0.34	0.93
		AG	0.43						
		AA	0.10						
*DGAT1*	rs409119650	TT	0.00	T C	0.10 0.90	0.18	0.82	0.16	0.49
		CT	0.19						
		CC	0.81						
*STAT5A*	rs161082816	GG	0.03	G A	0.14 0.86	0.24	0.76	0.21	0.34
		AG	0.22						
		AA	0.75						
*PRL*	rs422713690	GG	0.31	G A	0.53 0.47	0.50	0.50	0.37	0.26
		AG	0.44						
		AA	0.25						
*CSN1S2*	rs420391387	CC	0.22	C A	0.50 0.50	0.50	0.50	0.38	0.20
		AC	0.56						
		AA	0.22						
*GHR*	rs413776054	TT	0.05	T C	0.21 0.79	0.33	0.67	0.28	0.70
		CT	0.32						
		CC	0.63						
*GHRHR*	rs414991449	TT	0.43	T C	0.66 0.34	0.45	0.55	0.35	0.39
		CT	0.47						
		CC	0.10						

**Table 5:** Genetic parameters describing the SNP markers of the investigated candidate genes in the Barki lamb population.

Gene	SNP locus	Genotype	Genotypic Frequency	Allele	Allelic Frequency	He	Ho	PIC	HWE Test (*p* Value)
*LEP*	rs420693815	TT	0.34	T G	0.56 0.44	0.49	0.51	0.37	0.13
		GT	0.43						
		GG	0.23						
*IGF1*	rs400398060	GG	0.51	G A	0.70 0.30	0.42	0.58	0.33	0.54
		AG	0.39						
		AA	0.10						
*DGAT1*	rs409119650	TT	0.01	T C	0.07 0.93	0.13	0.87	0.12	0.68
		CT	0.13						
		CC	0.86						
*STAT5A*	rs161082816	GG	0.04	G A	0.22 0.78	0.34	0.66	0.28	0.44
		AG	0.37						
		AA	0.59						
*PRL*	rs422713690	GG	0.39	G A	0.61 0.39	0.48	0.52	0.36	0.56
		AG	0.45						
		AA	0.16						
*CSN1S2*	rs420391387	CC	0.38	C A	0.60 0.40	0.48	0.52	0.37	0.36
		AC	0.44						
		AA	0.18						
*GHR*	rs413776054	TT	0.01	T C	0.17 0.83	0.28	0.72	0.24	0.13
		CT	0.33						
		CC	0.66						
*GHRHR*	rs414991449	TT	0.49	T C	0.70 0.30	0.42	0.58	0.33	0.74
		CT	0.43						
		CC	0.08						

He: Heterozygosity, Ho: Homozygosity, PIC: polymorphic information content, HWE: Hardy–Weinberg equilibrium.

**Table 6:** Association of SNPs of studied genes with milk traits in Barki ewes (Mean ± SE).

Gene	SNP Locus	Genotype	Milk Yield (kg)	Fat %	Protein %	Lactose %	Total Solids %
*LEP*	rs420693815	TT (15)	35.55 ± 4.18	3.81 ± 0.32	5.33 ± 0.30	6.73 ± 0.24	19.17 ± 1.27
		GT (55)	26.67 ± 1.27	4.56 ± 0.26	5.24 ± 0.20	6.36 ± 0.22	19.05 ± 0.79
		GG (33)	29.77 ± 2.66	3.92 ± 0.20	4.67 ± 0.17	6.23 ± 0.17	17.24 ± 0.66
		*p* value	0.053	0.085	0.105	0.397	0.185
*IGF1*	rs400398060	GG (48)	29.59 ± 1.93	4.22 ± 0.22	5.06 ± 0.19	6.61 ± 0.19	18.93 ± 0.76
		AG (44)	28.46 ± 2.07	4.22 ± 0.26	5.14 ± 0.20	6.22 ± 0.21	18.39 ± 0.76
		AA (10)	28.08 ± 1.83	3.90 ± 0.39	4.35 ± 0.25	5.71 ± 0.18	14.56 ± 0.46
		*p* value	0.940	0.969	0.467	0.583	0.572
*DGAT1*	rs409119650	TT (0)	--	--	--	--	--
		CT (20)	26.25 ± 2.39	4.44 ± 0.28	4.94 ± 0.30	6.18 ± 0.33	18.43 ± 1.05
		CC (84)	29.56 ± 1.45	4.20 ± 0.19	5.04 ± 0.14	6.38 ± 0.14	18.26 ± 0.56
		*p* value	0.287	0.607	0.643	0.282	0.807
*STAT5A*	rs161082816	GG (3)	34.97 ± 4.02	3.45 ± 0.55	4.84 ± 0.81	4.19 ± 1.30	19.28 ± 3.41
		AG (21)	30.39 ± 2.83	3.84 ± 0.31	4.58 ± 0.22	6.38 ± 0.23	17.01 ± 0.86
		AA (73)	28.62 ± 1.52	4.37 ± 0.19	5.21 ± 0.16	6.52 ± 0.15	18.98 ± 0.60
		*p* value	0.638	0.297	0.149	0.001	0.199
*PRL*	rs422713690	GG (33)	32.59 ± 2.98	4.21 ± 0.24	5.09 ± 0.23	6.61 ± 0.22	18.38 ± 0.92
		AG (48)	25.90 ± 1.23	3.95 ± 0.24	4.93 ± 0.18	6.20 ± 0.20	18.02 ± 0.71
		AA (27)	30.62 ± 2.14	4.79 ± 0.35	5.40 ± 0.28	6.18 ± 0.30	19.96 ± 1.07
		*p* value	0.052	0.125	0.411	0.152	0.322
*CSN1S2*	rs420391387	CC (23)	31.44 ± 3.57	4.02 ± 0.32	5.05 ± 0.22	6.31 ± 0.25	17.56 ± 0.72
		AC (58)	28.61 ± 1.37	4.53 ± 0.25	5.12 ± 0.18	6.34 ± 0.19	19.56 ± 0.71
		AA (22)	25.33 ± 2.01	4.06 ± 0.30	5.09 ± 0.34	6.27 ± 0.31	17.47 ± 1.20
		*p* value	0.264	0.562	0.886	0.578	0.637
*GHR*	rs413776054	TT (5)	20.72 ± 2.77	4.93 ± 0.78	5.31 ± 0.37	6.51 ± 0.47	19.52 ± 2.53
		CT (32)	31.10 ± 2.57	4.25 ± 0.25	5.12 ± 0.21	5.98 ± 0.23	18.03 ± 0.86
		CC (64)	29.07 ± 1.50	4.20 ± 0.22	5.09 ± 0.19	6.49 ± 0.18	19.05 ± 0.68
		*p* value	0.249	0.556	0.791	0.450	0.667
*GHRHR*	rs414991449	TT (43)	28.61 ± 1.95	4.49 ± 0.26	5.32 ± 0.22	6.17 ± 0.23	19.26 ± 0.84
		CT (48)	29.71 ± 1.90	4.09 ± 0.25	4.96 ± 0.19	6.57 ± 0.19	18.71 ± 0.72
		CC (10)	26.44 ± 1.86	4.39 ± 0.35	5.12 ± 0.44	5.91 ± 0.44	16.76 ± 1.74
		*p* value	0.794	0.215	0.081	0.612	0.035

**Table 7:** Association of SNPs of studied genes with growth traits in Barki lambs (Mean ± SE).

Gene	SNP locus	Genotype	Birth Weight (kg)	Weaning Weight (kg)	Average Daily Gain (g)
*LEP*	rs420693815	TT (46)	3.67 ± 0.09	13.25 ± 0.60	106.0 ± 6.0
		GT (58)	3.75 ± 0.07	14.40 ± 0.52	118.0 ± 5.0
		GG (31)	3.82 ± 0.10	14.32 ± 0.61	117.0 ± 6.0
		*p* value	0.589	0.075	0.076
*IGF1*	rs400398060	GG (67)	3.75 ± 0.06	14.35 ± 0.42	118.0 ± 4.0
		AG (52)	3.73 ± 0.09	13.74 ± 0.60	111.0 ± 6.0
		AA (13)	3.63 ± 0.18	12.06 ± 0.92	93.0 ± 10.0
		*p* value	0.442	0.354	0.416
*DGAT1*	rs409119650	TT (1) ^@^	4.29	14.00	108.0
		CT (17)	3.86 ± 0.14	14.92 ± 0.62	123.0 ± 7.0
		CC (114)	3.71 ± 0.05	13.73 ± 0.38	111.0 ± 4.0
		*p* value	0.519	0.170	0.163
*STAT5A*	rs161082816	GG (4)	3.15 ± 0.28	11.23 ± 1.76	85.8 ± 19.0
		AG (41)	3.81 ± 0.11	14.67 ± 0.67	120.7 ± 7.0
		AA (66)	3.71 ± 0.07	13.86 ± 0.42	112.8 ± 4.0
		*p* value	0.631	0.255	0.273
*PRL*	rs422713690	GG (48)	3.75 ± 0.07	13.52 ± 0.45	109.0 ± 9.0
		AG (53)	3.74 ± 0.09	14.31 ± 0.59	117.0 ± 6.0
		AA (19)	3.70 ± 0.11	14.16 ± 0.82	116.0 ± 5.0
		*p* value	0.965	0.593	0.567
*CSN1S2*	rs420391387	CC (35)	3.79 ± 0.12	15.07 ± 0.75	125.3 ± 8.0
		AC (40)	3.86 ± 0.08	14.34 ± 0.48	116.5 ± 5.0
		AA (17)	3.72 ± 0.13	14.17 ± 0.63	116.1 ± 7.0
		*p* value	0.939	0.335	0.278
*GHR*	rs413776054	TT (1) *	3.5	20	183.3
		CT (35)	3.74 ± 0.11	14.77 ± 0.49	122.5 ± 5.0
		CC (70)	3.76 ± 0.07	14.31 ± 0.49	117.3 ± 5.0
		*p* value	0.608	0.446	0.309
*GHRHR*	rs414991449	TT (49)	3.72 ± 0.08	14.83 ± 0.58	123.4 ± 6.0
		CT (43)	3.79 ± 0.11	13.91 ± 0.58	112.4 ± 6.0
		CC (8)	3.73 ± 0.22	14.92 ± 0.81	124.4 ± 9.0
		*p* value	0.856	0.146	0.107

* Only one individual of the population carries the TT genotype.
